# The needs and preferences of Eastern Canadian sex workers in mitigating occupational health and safety risks through the use of Information and Communication Technologies: A qualitative study

**DOI:** 10.1371/journal.pone.0269730

**Published:** 2022-06-08

**Authors:** Thérèse Bernier, Amika Shah, Lori E. Ross, Carmen H. Logie, Emily Seto

**Affiliations:** 1 Institute of Health Policy, Management and Evaluation, Dalla Lana School of Public Health, University of Toronto, Toronto, ON, Canada; 2 Dalla Lana School of Public Health, University of Toronto, Toronto, ON, Canada; 3 Factor-Inwentash Faculty of Social Work, University of Toronto, Toronto, ON, Canada; 4 Women’s College Research Institute, Women’s College Hospital, Toronto, ON, Canada; 5 Centre for Gender and Sexual Health Equity, Vancouver, BC, Canada; Public Library of Science, UNITED KINGDOM

## Abstract

Sex workers may use Information and Communication Technology (ICT) as a means to mitigate occupational health and safety (OHS) risks by exchanging harm reduction techniques (e.g., screening) on blogs and social media. ICTs can also assist sex workers in creating online communities, where community members can act as each other’s safety check-ins, an additional harm reduction technique. In Canada, there is a paucity of research around ICT usage by sex workers for managing occupational health and safety. The objective of this study was to qualitatively examine the needs and preferences of Canadian sex workers when using ICTs in the delivery of strategies for occupational health and safety. Using a theoretical framework derived from a Social Ecological Model perspective, semi-structured interviews were conducted via phone, with a mixed gender sample (N = 22) of sex workers, between April and July 2020. OHS risks were found to be related to structural determinants, client behaviours, and lack of experience and knowledge when newly entering sex work. Participant accounts revealed a socially cohesive online community; however, sex workers reported difficulties in finding these communities, particularly when entering sex work. Such barriers to supportive communities were attributed to the criminalized, hidden nature of sex work that resulted in the fragmentation of harm reduction techniques across several online platforms, such as blogs, YouTube videos, closed electronic chat groups, and open online sex worker supportive communities. Moreover, these platforms and/or their content could potentially disappear without warning, either due to the platform provider seeking to evade possible prosecution, or because new legislation was introduced banning such content. Recommendations for further research include the co-design with sex workers of an innovative, secure, easily accessible, sex worker-only ICT OHS tool, utilizing a web hosting service located in a country where sex work has been either legalized or decriminalized.

## Introduction

Sex work, like many occupations, has its own unique set of occupational health and safety (OHS) challenges. Some of these challenges include violent clients, clients refusing to use condoms, harassment (either on the street or online), and the risk of their occupation being revealed to friends, family, and the general public (this risk is also known as “being outed”) [1–9]. These occupational challenges can be mitigated through harm reduction. In the context of sex work, harm reduction is a grassroots approach initiated by sex workers [10], who educate each other, exchange, communicate, and implement OHS strategies in order to reduce the potential harms associated with their work. In the context of this paper, sex workers use electronic Information and Communication Technologies (ICTs) as a means to mitigate these challenges. Advertising on websites and online marketplaces (e.g., craigslist, Backpages) facilitated the transition from outdoor street-based sex work to safer indoor workspaces [11]. ICTs provide sex workers with the means to screen clients as well as set the terms of the encounter in advance of the appointment with clients; this has led to a decrease in the rate of violence perpetrated against sex workers [12]. Sex workers use blogs and social media to facilitate the exchange of harm reduction techniques (e.g., screening) [11, 13, 14]. Since 2007, according to a study published by the Global Network of Sex Work Projects (nswp) in 2017, ICT use by sex workers, particularly the Internet and smartphones, has risen, reflecting the pace of growth in the general population [12]. While there has been an increase in studies on the use of ICTs by sex workers for managing OHS over the past five years [15], the knowledge around how to optimally leverage ICTs for this purpose remains limited.

Although ICTs present the opportunity to support the OHS of sex workers, censorship of Internet content related to sex work has made it more difficult for sex workers to communicate OHS strategies and share information on violent clients [11, 14, 16]. This form of censorship has also resulted in the loss of online sites that sex workers used to advertise their services. Without online advertising, finding clients may only become possible by going back to street-based, venue, or managed sex work. These types of avenues for sex work remove the sex worker’s ability to screen new clients before the encounter and rely on third party intermediaries who may or may not have the sex worker’s safety in mind [11, 14, 16]. On April 11, 2018, the United States (U.S) Congress passed two laws, the Fight Online Sex Trafficking Act (FOSTA) and the Stop Enabling Sex Traffickers Act (SESTA), collectively known as FOSTA/SESTA, that prohibited sex work content on websites, including advertising and harm reduction techniques [11, 13, 14]. While FOSTA/SESTA is specific to the U.S., its governance of the U.S.-based social media platforms (Twitter, Facebook, Instagram) has had implications on sex workers worldwide. Sex workers have reported that community support groups on Facebook were removed without warning by Facebook, although no solicitation was taking place in those groups [11]. Sex workers report that they have been deplatformed (also known as removed) from Instagram [11, 17, 18] and Twitter [11, 17–19], although Twitter denies they engage in this practice [17]. Sex workers report experiencing “shadowbanning”, an algorithmic curation technique that prioritizes whom an individual sees in their list of “following” on Twitter and “friends” on Facebook [11, 20]. Twitter also denies they shadowban accounts [19]. The practice of shadowbanning prevents sex workers from seeing other sex workers in their social media feed, which could result in a loss of information regarding violent clients or other safety information [11]. In Canada, where this study took place, the law that governs sex work, Bill C-36 (The Protection of Communities and Exploited Persons Act [PCEPA], now law since December 6, 2014), criminalizes the advertising of sexual services [3, 21]. PCEPA also criminalizes the communication of information related to sex work, whether verbally, in print (hard copy), or electronic format. Moreover, anyone found distributing such information faces the possibility of criminal prosecution for making such information available [5, 22, 23]. Nevertheless, sex workers continue to utilize all forms of communication to ensure their safety.

Research around ICT usage by sex workers has taken place in India (8 studies), Australia (5 studies) and the UK (5 studies), resulting in 18 studies in total [15]. Comparatively, in Canada, there is a paucity of research around ICT usage by sex workers, especially as applied to Eastern Canada [5, 9, 23, 24]. This articles examines the needs and preferences of sex workers in Eastern Canada when using ICTs in the mitigation of OHS risks.

## Method

### Study design

OHS frameworks documented in the literature are relevant to formal workers protected by OHS legislation. However, these frameworks do not apply to Canadian sex workers, as they are considered informal workers and the law (PCEPA) criminalizes their work. For this research project, a theoretical framework derived from McLeroy’s Social Ecological Model [25] assisted the researchers in viewing the OHS strategies adopted by sex workers, and the methods used to communicate these strategies among peers and colleagues. The model examines levels of influence on OHS risks as they are experienced by sex workers: intrapersonal (knowledge, skills); interpersonal (formal and informal support); community (sex worker collectives); structural determinants (legal, social and political); and the interaction among the levels that drive OHS and strategies to manage OHS. Guided by the Social Ecological Model [25], approximately 20 semi-structured questions were developed covering OHS, social networks, electronic devices, and privacy. The research project was approved by the University of Toronto’s Health Science Research Ethics Board protocol #37920.

### Setting and sampling

For this research project, the community-based research (CBR) approach was employed. CBR has been influenced by the work of Paulo Freire [26]. His research approach embodies these principles: participant inquiry and that the participants in the community have the knowledge to solve their issues and transform their circumstances [27]. A research design using a community-based research approach involving sex workers is consistent with the guidelines published in 2012 by the World Health Organization with UNAIDS and the Global Network of Sex Work Projects (nswp) which recommend scaling-up structural interventions that enhance sex worker-led community empowerment [28]. Prior to beginning this research project, a community-academic partnership was established with Maggie’s: Toronto Sex Workers Action Project (Maggie’s), a community-based organization (CBO) advocating for sex workers’ rights [29]. In keeping with CBR tenets, an advisory committee was formed, composed of the interim Executive Director, the program manager, and a peer support worker. In their role as local stakeholders, the advisory committee has provided support in learning to navigate the culture of the sex worker community. The advisory committee also reviewed the interview guide (no changes were requested). Members of the advisory committee have been kept apprised of the progress of the research, through regular updates via e-mail.

Sex workers were invited to participate in the research if they met the following inclusion criteria: aged 18 years or older, able to speak English, working in Canada, have worked or were currently working in sex work, and possessed a cell phone capable of receiving text messages and/or accessing a website, Facebook, and/or Twitter.

A multi-pronged strategy was used to recruit sex worker participants. A presentation on the research project at Maggie’s premises by the lead author (TB) (pre-COVID-19 pandemic) resulted in engaging 7 participants via purposive sampling [30]. Purposive sampling continued through these methods: onsite at events produced for sex workers (pre-COVID-19 pandemic) resulting in 4 participants; social media invites resulting in an additional 4 participants. Snowball sampling [31] was also used to invite participants. A call out to a former sex worker known to TB resulted in the participation of 4 men sex workers. A purposive participant contacted on social media referred 2 of their colleagues. Contact with a peer support worker at a community-based organization in London, Ontario resulted in 1 participant. Through purposive and snowball sampling, 22 participants were invited to the research project, all located in Eastern Canada (Ontario, Québec, and Nova Scotia). All participants gave their informed written consent and were compensated CAD$30 per hour for their participation.

### Data collection and analysis

Phone interviews, ranging from 40 minutes to 2 hours in length, were conducted by the lead author (TB) between April and July 2020. All interviews were audio-recorded and stored on a secure University of Toronto server accessible solely by TB. Interviews were transcribed verbatim by a professional transcription service. Transcripts were subsequently imported into NVivo version 12 (QSR International, Chadstone, Australia) [32], a qualitative data analysis software package, to manage coding of the transcripts by two authors (TB and AS).

The qualitative analysis proceeded using Braun and Clark’s thematic content analysis approach [33]. TB and AS independently analyzed seven randomly selected transcripts. Two types of analysis were conducted, open coding and in vivo coding, with each type of code, once detected, stored in NVivo, supplemented by its associated fragment of text [34]. TB and AS then compared each other’s coding, resolved differences, and agreed on a common set of codes. Using the common set of codes, a further seven transcripts were analyzed independently, this analysis did not preclude the eventuality of identifying new codes. Another round of reviewing and agreement on codes took place. In this second round, TB and AS agreed to categorize the codes into a broader thematic framework based on the constructs in the Social Ecological Model [25]. The first 14 transcripts were re-coded and the remaining 8 transcripts were coded with the agreed-upon scheme. Memoing (writing up findings concurrently as the analytical process progressed [34]) was performed by both TB and AS; journaling was performed by TB for reflexivity after each interview; AS performed journaling after reading through each interview transcript [34]. Both of these practices yielded additional rigour during the analytic process.

## Results

### Demographic profile

Drawing on phone interviews with a mixed gender sample (N = 22) of sex workers, participants ranged in age from 20 to 53 years old (33.55 years on average), with a range of 1 to 32 years in sex work. In terms of gender, participants identified as either cisgender women, cisgender men, trans women, or non-binary. In terms of sexual orientation, participants identified as either bisexual, gay, heterosexual/straight, or pansexual/queer/fluid. Sex workers performed a variety of sex work, including escort (independent, without an agency), working online, camming, dancer, phone operator, massage, and dominatrix. The demographic profile of participants is available in Table 1.

**Table 1:** Demographic profile.

	*N = 22*
**Age (years): mean, range**	33.55, 20–53
**Number of years in sex work: mean, range**	7.36, 1–32
**Gender identity**	
Cisgender man	4
Cisgender woman	14
Non-binary	1
Trans woman	3
**Total**	22
**Sexual orientation**	
Bisexual	8
Gay	4
Heterosexual/Straight	2
Pansexual/Queer/Fluid†	8
**Total**	22
† *summed to protect participants’ privacy*	
**Type of sex work**	
Indoor/Escort^1^	21
Outdoor (street-based)^2^	1
Online^3^	11
Camera (Cam)^4^	10
Dancer^5^	5
Phone operator^6^	2
Massage^7^	3
Dominatrix^8^	1
* *a sex worker may perform more than one type of sex work*	

^1^ An indoor sex worker is a worker that receives clients at their home or goes to the client’s location. All escorts interviewed worked independently, without an agency

^2^ A street-based sex worker is a worker that transacts with clients outdoors

^3^ An online sex worker is a worker that uploads pictures or videos of themselves to a website dedicated to this purpose, for example, OnlyFans

^4^ Camming is similar to an online sex worker, in this case, the sex worker uploads their pictures or videos to their own websites

^5^ This work is performed in a club setting, the worker dances to music while removing their clothing, not necessarily all of their clothing

^6^ A person that engages in sexually stimulating conversations with a client

^7^ A person that gives massages to a client, usually in a venue dedicated to this type of work

^8^ A dominatrix is engaged by a client who enjoys being restrained

### Thematic analysis

From participants’ accounts, the following broad thematic areas were identified: (1) the landscape of ICT use by sex workers in Eastern Canada, (2) the OHS risks associated with sex work, (3) current use of ICTs to mitigate OHS risks, and (4) the potential use of ICTs for OHS risk mitigation.

#### 1- The landscape of ICT use by Sex Workers in Eastern Canada

Sex workers reported the importance and utility of ICTs for their business activities. All 22 participants reported ownership of a smartphone, with some sex workers having a separate phone for their work, and a separate phone for personal use. Texting was the most often used method of communication with clients. Sex workers expressed that the camera on their smartphone allowed them to take high quality pictures and videos, which became especially important during the COVID-19 pandemic when their work had moved online to sites where people paid to view their photos (e.g., OnlyFans [35]). In addition to their smartphone, all interviewed sex workers owned at least one of these devices: tablet, laptop, or desktop. Sex workers explained that the larger screen size and availability of a keyboard on laptops and desktops made some aspects of their business activities easier, such as posting ads, creating and editing videos (camming), checking their social media accounts, and maintaining their websites.

“*Smart phone or laptop*, *your two best friends in this industry*. (…) *because I’m an indoor worker*, *we live by them*. *It’s how we create our clienteles*, *it’s how we build our business*. *It’s not like we do a lot of public street work kind of idea*. *So*, *it’s a different avenue to go through*.” (I-SW21)

While smartphones and laptops were used on a daily basis to access the Internet and social media, sex workers referenced the digital divide [36] as they acknowledged that it was a privilege to own ICT devices and to afford data plans that enabled this access.

“*a lot of my friends aren’t wealthy enough to have data plans* (…) *would just be the texting and calling*, *if you don’t have a data plan*” (I-SW1)

Although having ICTs as business tools were advantageous, sex workers expressed that using smartphones and other ICTs brought upon new risks. Sex workers highlighted that the constant availability of their online workspace led them to continuously work (e.g., checking responses to their ads on multiple platforms), often at the expense of their own physical and mental health. To counteract the risks of occupational burnout when working online, sex workers reported that they learned to establish boundaries around their work schedule.

“*one of my issues being independent early on was*, *I just was on my phone all day*, *working and keeping up* (…) *you have to figure out a balance and if you’re working on your phone*, *you can always be working*.” (I-SW2)

Sex workers recounted additional risks when using smartphones and other ICTs. Sex workers explained that the risk of “outing”, or being exposed as a sex worker, is present due to having an online presence. Sex workers also spoke about the risk of arrest due to having a smartphone that records texts sent and received, as well as locations frequented, as the phone becomes a de facto diary.

“*It can be used as evidence*, *do you know what I mean*? *You know*, *because if you’re text-messaging*, *you know what I mean*, *clients*, *you’ve got to be mindful of*, *you know*, *making sure you don’t have a call log*, *you don’t have a text message log* (…) *the smartphone*, *as much as it has a lot of positives*, *it’s also now being used against us to criminalize us*.” (I-SW7)

#### 2- OHS risks associated with sex work

The sex workers outlined several risks associated with their occupation, which fell under three categories: structural determinants, clients, and entering sex work.

**Structural determinants.** Although participants expressed that they would like to work together out of a shared location, the laws in Canada prohibit this type of work environment. Sex workers noted that criminalization not only isolated them physically from other sex workers, but also prevented them from communicating safety information to each other through verbal, paper, or online modes. Sex workers talked about websites and social media platforms that had removed sex worker content after the passage of FOSTA/SESTA in the United States.

“*they keep shutting down all of our safety sites*, *like all of the sites we used to talk to each other and find this* [OHS] *information*.” (I-SW22)

Sex workers related that in the context of criminalization, the police were not considered a resource for their protection but rather an added risk to their safety and wellbeing. In case of assault, robbery, or harassment, sex workers reported that they would not, or be very reluctant to, contact the police, as police will not arrest the client, instead, they will arrest the sex worker.

“*it’s very challenging for a lot of sex workers to contact police if they’re*, *you know*, *experiencing abuse*, *like when they’re doing their work because … because it’s highly criminalized*, *and a lot of sex workers are not out*. *It’s scary*. *And a lot of times police don’t care*. *They don’t take it seriously*. *They discriminate against sex workers*.” (I-SW6)

Sex workers mentioned that working in a criminalized industry deprived them of formal labour protections, and they were aware that their occupation was viewed as having no merit.

“*the stigma associated with sex work and the laws that are in place that make it so that we can’t fight for our rights within the workplace and our rights to safety*.” (I-SW17)

Sex workers recounted the poor attitudes, stigma, and discrimination they faced when accessing health services, which prevented them from being open about their occupation and made seeing a health professional anxiety provoking. The participants noted that such attitudes towards sex work also permeated throughout their personal lives, often leading them to lie or keep their work hidden from their friends and families. Sex workers also discussed the marginalization they experience from having their work stigmatized, and that this feeling of marginalization prevented them from seeking out a supportive community.

“*the laws that are supposed to protect us*, *and they really just cause further damage and ostracize us*, *and push us to a place where*, *you know*, *like we have to*, *you know*, *lie about what we do to be able to get a house* (…) *the law influences how the rest of the world perceives us*, *and that includes accessing social services*, *that includes accessing healthcare*, *that includes accessing housing*.” (I-SW17)

**OHS risks with clients.** Sex workers indicated that the most pressing OHS concern they faced, regardless of their gender, was violence. Clients, whether new or recurring, could become violent, particularly if they were intoxicated. Sex workers recounted the types of violence that they had experienced, for example, sexual assault. One participant recalled a particular act of violence, confinement, where they had accepted an outcall [a location other than the sex worker’s home], and the client then proceeded to confine the sex worker to the client’s premises. Sex workers also discussed the risk of acquiring sexually transmitted blood-borne infections (STBBIs) [37]. Participants explained the risk of stealthing, another form of violence, where a client either attempted to remove the condom or completely removed the condom during a sex act. To minimize these risks during an appointment, some sex workers mentioned employing strategies such as a no drugs and alcohol policy and mandating condoms during appointments.

“*I was*, *like*, *you know*, *I said*, *I thought so*. *I said*, *dammit*, *that’s come off*. *And he went*, *I thought it felt better*. *And I was like*, *oh*, *you knew and you said nothing*? *So we did have a talk about it*, *and I said to him*, *I said*, *that’s not cool*. *And he said*, *yeah*, *but you get screened*. *I said*, *that’s not the point*, *not the point*.” (I-SW18)

**Entering sex work.** Several participants discussed that they were not aware of best OHS practices in the industry, such as screening clients and setting boundaries, when they first engaged in sex work. Early in their career, sex workers recalled that they booked appointments made solely on the basis of one text or e-mail from the client, without further screening the clients. Some sex workers recounted a lack of knowledge around consent and establishing boundaries (e.g., services provided, requiring use of condoms) either when they were setting up the appointment or once they were in the room with the client. Sex workers reported difficulty in accessing resources that would help them understand safety in sex work and that the current laws prevented them from operating safely.

“*I would only have to trust my gut feeling over a few texts or emails*. *And that didn’t seem safe for someone who didn’t really know what she was doing and had never done it before* (…) *what happens in the room you learn on your own*. *Like you learn how to enforce your boundaries*.” (I-SW15)

### 3- Current use of ICTs to mitigate OHS risks

**Obtaining OHS information.** The Internet was perceived by participants as an essential source of information, a conduit into connecting with an online community. Sex workers reported using Google to search for sexual health information, legal information, safety concerns for escorts, and sex work in general. Sex workers mentioned that they found community-based organizations (CBOs) that were run by and for sex workers, and for the first time in their sex work career, accessed bad date lists on the CBO’s website. Escort review boards, blogs written by sex workers, and sex worker websites were also useful sources of information identified by participants. Sex workers also recounted using social media platforms such as Facebook groups, Reddit, YouTube, and Twitter, and through these platforms, networked with the more experienced sex workers they found online.

“*you really just got to use Google*, *YouTube*, *try to get on Twitter*, *talk to other girls*, *find out what they’re doing to keep themselves safe*.” (I-SW19)

**Screening.** Sex workers reported that they used screening to determine whether it was safe to meet a client who found them through their ads or website postings. Participants explained that their ads contained very specific protocols, such as how to contact them (e.g., text only, no phone calls, e-mail only), where the appointment will take place (e.g., no in-calls [an in-call is defined as meeting a client in the sex worker’s home]), as well as terms of the encounter (e.g., no activities without condoms, no drugs or alcohol). Clients who did not follow the outlined protocols would be refused, while those following protocols would be further screened, for example, based on their use of respectful language. Sex workers indicated that screening helped them ascertain whether their boundaries would be respected, and whether they would be physically and emotionally safe during the encounter.

“*that goes back to the initial screening*, *where I’ve had text conversations and I’ve got a feel*. *So some people behave in certain patterns*. (…) *it is*, *in my opinion*, *very important for me to have a feel for who’s coming*.” (I-SW18)

**Bad date lists.** Sex workers accessed bad date lists from several sources: locally, from CBOs; escort review boards, which are either local or Canada-wide; or lists available for a fee. Other sex workers reported exchanging bad date information through Twitter, group chat, or Google sheets. When encountering a disrespectful or violent client, sex workers described that they would block the client on their phones, report the client to a bad date list and inform their network of colleagues. Sex workers agreed that bad date lists had kept them from seeing a potentially violent client.

*“Because they give me a better sense of security around who I’m going to see*. *Bad date lists have saved me from seeing potentially violent clients in the past and they’re especially vital I find when you’re a new worker because those are the workers that predators tend to target because they don’t know about these long-standing predators that workers who’ve been around for a while are familiar with already*. (I-SW22)

Although bad date lists were a valuable OHS tool, not all sex workers used them, due to accessibility issues. Some sex workers explained that if they did not have access to a bad date list, they tapped into their network to obtain information about a client. Some sex workers shared that they were not aware of the existence and availability of bad date lists. One sex worker described that online bad date lists can only be accessed on a device with Internet access. Sex workers described that to have access to a bad date list, the sex worker had to be vouched for by another sex worker that already had access to the list. Sex workers agreed that vetting access helped prevent clients from scanning bad date lists.

“*the thing about a blacklist is you want it to be kept secret*. *You know you don’t want clients to have access to it because if a client figures that he’s on the blacklist he can just change information* (…) *you have to make sure that it’s something that’s secure*. *And that it won’t be outed and that’s something that’s always a little harder online and especially with a group of a lot of people*.” (I-SW15)

**Buddy system.** Some sex workers explained that prior to meeting a client, they text a group of friends or colleagues, using WhatsApp, Twitter, or other electronic messaging software, to apprise their network of their location and scheduled end time of the encounter. This OHS strategy was referred to as a safety call, buddy system, or check-in group.

“*I also started up the one Twitter group that I said*, *we have the group chat*, *that group is a check-in group as well*.” (I-SW17)

**Online communities.** Sex workers have found online communities in community-based organizations (CBOs) websites that were run by and for sex workers. Sex workers also described finding online communities on escort review boards, blogs written by sex workers, and sex worker websites, as well as social media platforms such as Facebook and Twitter. Sex workers described that the strong online communities they had created provided support, kept each other physically safe, helped each other manage their mental health, and together fought the stigma imposed upon them by social norms.

“*I was so grateful that I found the community and how helpful it’s been*, *because we really protect each other*, *because otherwise*, *you know*, *we don’t survive* (…) *technology has really changed the way we network and take care of each other that way*, *I would say*, *because yeah*, *it’s–and you just put a trigger or content warnings about whatever you’re going to talk about*, *so–If you want to narrow it down*, *I would say*, *in terms of technology*, *that definitely helps in terms of processing and getting the support that we often really can’t get elsewhere* (…) *I’m also a moderator of a mental health group chat*, *and I’m also in a group chat for just general support for Toronto*, *mostly escorts*.” (I-SW2)

Table 2 depicts the usage by sex workers for each type of ICT device.

**Table 2:** ICT device and purpose.

ICT device/ Purpose	Smartphone	Tablet	Laptop	Desktop
Phone call	Screening			
Texting	Screening; Bad date list; Buddy system		Screening; Bad date list Buddy system	
WhatsApp	Screening; Bad date list; Buddy system			
Telegram	Community			
TextNow	Community			
Facebook (FB)	Community			Community
FB messenger	Community; Bad date list			Community; Bad date list
Instagram	Community			
Twitter	Community; Bad date list Buddy system		Community; Bad date list; Buddy system	
E-mail	Screening	Screening	Screening	Screening
Video calling		Screening		
Google sheets			Bad date list	
Information searches	OHS information		OHS information	OHS information
Blogs	OHS information	OHS information	OHS information	OHS information
listservs	OHS information		OHS information	
Signal	√			
Reddit	√			√
Podcast	√			
Streaming		√	√	
Dating app	√			
Google calendar	√		√	
Edit photos and video			√	
Camming			√	
Post ad/check ad		√	√	√
Website maintenance				√
Data storage				√

### 4- Potential use of ICTs for OHS risk mitigation

Although sex workers described how their current usage of ICTs assisted them in managing the OHS risks associated with sex work, they also highlighted the limitations of their current use, including challenges associated with finding other sex workers online and the prospect that safety information may be taken down due to local regulations and legislation related to sex work content on the Internet.

“*there is more resources that are coming about*, *and the thing is*, *is that with the laws in place*, *it’s technically a liability*. *(…) you can’t really just put out a list on how to screen*, *you know*, *that’s something we have to do privately together*. *(…) So I’m very hopeful that in the future that we’ll be able to access to more resources and tools like that*, *but it’s one of those things where it’s like there are people putting forth laws right now that are making that hard*.” (I-SW17)

The participants in this study, regardless of gender, envisioned an innovative, secure, easily accessible, sex worker-only ICT OHS tool to address these limitations.

“*I think it would be invaluable*. *I don’t think I can out into words just how invaluable that would be*. *(…) a strategic and well-thought-out and easy to access platform*, *would be wonderful*. *And something that’s interactive*. *Interactive to me means everyone can access it*, *you know*, *a login or a password provided for sex workers*, *a verification process*.” (I-SW18)

Their specific recommendations are further articulated in Table 3. These recommendations were not specific to one gender. During the interviews, all genders mentioned that having OHS information available when entering sex work would have been helpful. Access to bad date lists and connecting with other sex workers to form a buddy system were two more requirements mentioned in a potential digital OHS tool. Participants also mentioned that if the source of the information was other sex workers, there would be a higher level of trust in the information.

**Table 3:** Participant recommendations for ICTs for OHS risk mitigation.

ICT recommendation	Illustrative quote
A website that is a central repository of OHS information, accessible via any type of computer device	*“It would be fantastic to be able to access all of those resources in one place*. *I totally think that would be a really helpful resource to have accessible online*.” (I-SW14)
A section of the website would be dedicated to universally accessible bad date lists, with links to local, provincial and national bad date lists, a forum where sex workers could discuss their experiences with these dates, and a look-up phone number feature	*“A comprehensive bad date list that everyone could access whether or not they’d be indoors*, *whether they’re on the street*, *whether they’re in massage parlors”* (I-SW22)
The website would comprise a list of resources of trusted healthcare providers, therapists, STI testing clinics, as well safe spaces to work (hotels and apartments), and legal clinics	“*Having lists of*, *like*, *local social workers would be a great idea*, *you know*, *accountants that are sex worker friendly*, *massages that are sex worker friendly*, *doctors that are sex worker friendly*, *clinics*, *all of that information in one would be so helpful*.” (I-SW19)
Sex workers could also connect with each other and form buddy systems	“*Reaching out to other sex workers that you trust*, *or that you’re friends with*, *to develop some kind of buddy system*.*”* (I-SW9)
Sex worker communities would come together on forums and chat groups to offer support	*“I think having community forums*, *like closed groups and like Twitter where people can share resources and get advice from each other is really important*. *I think also having access to community groups like Maggie’s*, *like Butterfly [sex work] groups where you can also access those support workers to talk to and to get resources from online is really important”*. (I-SW13)
A page dedicated to keeping sex workers updated on laws would also be available	*“Keeping sex workers updated on laws that are*, *you know that are*, *that are changing; protocols that are changing in a sense of being criminalized more*. *(…) I think it’s important that we’re always updated on stuff like that*. *So*, *we’re informed*, *and we can inform others and clients*.” (I-SW7)
As working online carries a risk of outing and other privacy violations, information on using technology safely would also be available	*“The IT thing is a really important point of view because you know there are so many gaps I think in like technology that sex workers could need or use or even help innovate (…) I’m always concerned around how is that information going to be used*? *How is it going to be protected from like hackers and shit like that*.*”* (I-SW22)
Source of the OHS information on the website	*“That whoever’s putting this together is someone who is a sex worker who is working with other people*. *Like there are sex workers on the team somewhere*. *(…) Outsiders like they have good intentions*, *but they may not think things through in the same way that a sex worker would*. “(I-SW22)
Granting access to the website	*“The vetting system would have to be–there’d have to be some checks and balances in the vetting system*. *Like*, *some groups on Facebook–like*, *I’m in a couple of groups where*, *like they ask a question*, *as an entry question*. *Do you believe that sex work is valid work*?*”* (I-SW4)

## Discussion

The results of this study provide insights into the usage of ICTs by Eastern Canadian sex workers to manage their OHS risks. Guided by the Social Ecological Model [25], this study found that a sex worker’s electronic OHS toolkit was comprised of screening procedures, search results from bad date lists, a buddy system, and a supportive online community. Importantly, the smartphone was identified as the preferred ICT for sex workers, as all participants interviewed possessed a smartphone. ICT usage by sex workers may help to reduce the risk of violent encounters with clients, and may facilitate connections to an online community, thus alleviating social isolation and stigma.

As reported in previous research [5, 6, 38–40], the risk of violence during an encounter was a central concern across sex workers of all genders in this study. To minimize the risk of violent clients, sex workers reported using rigorous screening protocols at the time of booking to assess the risk of seeing a client [9, 41]. Some participants in this study also used electronic bad date lists to view which clients posed a danger during an encounter, as an additional step in their screening process. As working in a criminalized occupation often prevented sex workers from contacting police services to report violence and harassment either for fear of arrest or dismissal [42–48], bad date lists served as an alternative means of reporting violent acts committed by clients. However, electronic bad date lists are not universally accessible, as not all sex workers possess the technology required to access them. In addition, while accessing formal bad date lists through a vetting process is a safety measure to ensure the accuracy of the lists, it creates another barrier to access for sex workers with limited connections to sex worker communities.

Although previous studies have found that transitioning to indoor online work has isolated sex workers from each other [5], this study found that participants had created socially cohesive online communities by exchanging harm reduction techniques on blogs, websites and social media. These communities, whether formal through a CBO, or informal groups that grew organically through networking, seemed effective in enabling the same kind of social cohesion experienced among sex workers operating in physical spaces [49, 50]; for example, having a peer to talk to about their mental health, or willingness to share their expertise. Despite the value of these online communities, sex workers reported difficulties finding them, particularly when first entering sex work. Barriers to finding and accessing supportive communities were attributed to the criminalized, hidden nature of sex work that resulted in the fragmentation of communities across a number of online platforms. Moreover, sex worker-themed platforms were not always accessible due to legislative constraints that prevent sex workers from accessing, gathering or distributing online information related to sex work, and the possibility of criminal prosecution for making available such information [5, 22, 23]. This could be addressed by creating online sex worker communities utilizing a web hosting service located in a country where sex work has been either legalized (e.g., Switzerland, Germany [41]) or decriminalized (e.g., New Zealand [51]).

Participants in this study mentioned the impact of sex work stigma on their OHS risks, consistent with the risks identified in Benoit et al.’s 2018 study on prostitution stigma. Notably, participants described how judgement on the part of healthcare workers could result in avoidance of health care services [40], as has been documented in previous studies [4, 52, 53]. To address this concern, the participants in this study recommended a directory of professionals that do not stigmatize sex workers, including healthcare providers, therapists, and STI testing clinics, be made available as part of a novel ICT OHS tool.

Finally, a community has been found to help in learning harm reduction techniques for sex work [11]. Individually, the participants in this study identified an array of strategies they use for OHS and stated their willingness to share their expertise with other sex workers. Further research with sex workers as the designers of ICT OHS tools needs to be undertaken to continue expanding on the social capital already present in online sex worker communities [11]. Such ICT tools offer opportunities to share the OHS benefits derived from participating in these communities, and at the same time, design methods to dismantle identified barriers to access.

## Limitations

This study has several limitations. A limitation related to the COVID-19 pandemic is that the study did not include sex workers who hid their occupation from their housemates and therefore could not openly speak on the phone about their experiences with ICTs. Two other limitations of the study are that only a small number of transgender sex workers participated, and only one street-based sex worker was interviewed, limiting the transferability of our findings to these groups. Lastly, sex workers in urban centres who use ICTs in their work were interviewed, excluding sex workers in rural and remote areas, as well as those without ICTs. Future research will be needed to ensure that any ICTs developed as a result of these recommendations are also of use to sex workers in rural and remote settings.

## Conclusion

This study adds to the emerging research that examines, in collaboration with sex workers, the use of ICTs in their work, and more specifically, the use of ICTs in the delivery of strategies for OHS. Despite the legislative constraints imposed on sex workers with regards to communicating safety information, either in physical or in online spaces, sex workers continue to make creative use of the ICTs available to them. However, these constraints result in OHS information being scattered across various platforms such as blogs, YouTube videos, closed electronic chat groups, and open online sex worker supportive communities, creating access challenges, particularly for individuals new to sex work. Moreover, these platforms may disappear without prior warning, either due to the platform provider seeking to evade possible prosecution, or because new legislation is introduced banning such content. Innovative, secure, easily accessible, sex worker-only ICT OHS tools, designed by sex workers for sex workers, may assist in reducing the on-the-job risks sex workers face on a daily basis, until legislative changes are implemented which reduce the OHS risks associated with criminalization.
